# Preliminary Study on New Alternative Binders through Re-Refined Engine Oil Bottoms (REOBs) and Industrial By-Product Additives

**DOI:** 10.3390/molecules26237269

**Published:** 2021-11-30

**Authors:** Michele Porto, Paolino Caputo, Valeria Loise, Abraham A. Abe, Giulia Tarsi, Cesare Sangiorgi, Francesco Gallo, Cesare Oliviero Rossi

**Affiliations:** 1 Department of Chemistry and Chemical Technologies, University of Calabria, Via P. Bucci, Cubo 14/D, 87036 Rende, Italy; michele.porto@unical.it (M.P.); abraham.abe@unical.it (A.A.A.); cesare.oliviero@unical.it (C.O.R.); 2 Department of Civil, Chemical, Environmental and Materials Engineering, Viale del Risorgimento 2, 40136 Bologna, Italy; giulia.tarsi2@unibo.it (G.T.); cesare.sangiorgi4@unibo.it (C.S.); 3 Itelyum Regeneration Srl, Via Tavernelle 19, Pieve Fissiraga, 26900 Lodi, Italy; francesco.gallo@itelyum.it

**Keywords:** circular economy, waste materials, REOB, alternative binders, asphalt mixture

## Abstract

Recent studies have worked towards addressing environmental issues such as global warming and greenhouse gas emissions due to the increasing awareness of the depletion of natural resources. The asphalt industry is seeking to implement measures to reduce its carbon footprint and to promote sustainable operations. The reuse of several wastes and by-products is an example of a more eco-friendly activity that fulfils the circular economy principle. Among all possible solutions, the road pavement sector encourages, on one hand, the use of recycled materials as a partial replacement of the virgin lithic skeleton; on the other hand, it promotes the use of recycled materials to substituting for a portion of the petroleum bituminous binder. This study aims to use Re-refined Engine Oil Bottoms (REOBs) as a main substitute and additives from various industrial by-products as a full replacement for virgin bitumen, producing high-performing alternative binders. The REOBs have been improved by utilizing additives in an attempt to improve their specific properties and thus to bridge the gap between REOBs and traditional bituminous binders. An even larger amount of virgin and non-renewable resources can be saved using these new potential alternative binders together with the RAP aggregates. Thus, the reduction in the use of virgin materials is applied at the binder and the asphalt mixture levels. Rheological, spectroscopic, thermogravimetric, and mechanical analysis were used to characterize the properties, composition, and characteristics of the REOBs, REOB-modified binders, and asphalt mixes. Thanks to the rheological investigations of possible alternative binders, 18 blends were selected, since they behaved like an SBS-modified bitumen, and then they were used for producing the corresponding asphalt mixtures. The preliminary mechanical analysis of the asphalt mixtures shows that six mixes have promising responses in terms of stiffness, tensile resistance, and water susceptibility. Nevertheless, the high variability of recycled materials and by-products has to be taken into consideration during the definition of alternative binders and recycled asphalt mixtures. In fact, this study highlights the crucial effects of the chemical composition of the constituents and their compatibility on the behaviour of the final product. This preliminary study represents a first attempt to define alternative binders, which can be used in combination with recycled aggregates for producing more sustainable road materials. However, further analysis is necessary in order to assess the durability and the ageing tendency of the materials.

## 1. Introduction

An ever-increasing pressure for resources conservation and environmental protection—e.g., CO_2_ reduction [1]—has led to a systemic change in the use and recovery of resources towards a clear transition to a regenerative circular economy [2,3]. This has been done by creating a closed-loop system, minimizing the use of resource inputs and the creation of wastes, pollution, and carbon emissions [3]. New potential pathways in innovation and investment, reducing wastes and promoting the continual use of resources, have therefore been proposed [4,5]. Considering this aspect, waste ceases to be waste and acquires the status of End of Waste product (EoW). The End of Waste (EoW) criteria include recovery and treatment processes under which waste could be converted in a new potential product. In particular, according to the European standards [6], the main requirements to satisfy the EoW criteria for a given waste (possibly treated by industrial processes) are: (a) the substance or object is intended to be used for specific purposes; (b) there is a market or demand for this substance or object; (c) the substance or object meets the technical requirements for the specific purposes and complies with the existing legislation and standards applicable to the products; (d) the use of the substance or object will not lead to overall negative impacts on the environment or human health (in accordance with the Substance of Very High Concern (SVHC) list [7]).

From this perspective, the reuse of re-refined exhausted oils from vehicles and industrial hydraulic applications [4,5] that have become unfit for the use for which they were originally intended completely fulfils circular economy goals. In the view of a circular economy and considering the opportunity to reduce as much as possible the extraction of crude oil, a study has been started between universities and industries [8,9,10,11] that aims to produce new potential alternative binders (or eco-binders) by using recycled materials and/or by-products. In detail, the present research used Re-refined Engine Oil Bottoms (REOBs) to obtain suitable petroleum-based binders, enhancing the properties of these by-products, which are mainly used as bitumen-like product or bituminous membrane additive. The physical properties of the REOBs were enhanced by using a set of synthetic and/or natural additives such as powdered rubber from End-of-Life Tyres (ELTs) or other waste polymers [12], cellulose from waste paper or from olive pomace wastes, other suitable wastes or industrial by-products, and cheap chemical products. The incorporation of additives into REOBs aims to create alternative binders that could be used for producing asphalt concretes whose mechanical properties satisfy the technical requirements. Moreover, Reclaimed Asphalt Pavement (RAP) aggregates were used to obtain the asphalt concrete samples that underwent testing in order to study their behaviour with the goal of substituting the virgin aggregates, which are commonly employed in asphalt pavement. This would allow for meeting the standards of many European countries where RAP is already re-introduced in Hot Mix Asphalt (HMA) and Warm Mix Asphalt (WMA) mixes in the range of 70–90% by the total weight of available RAP material [13]. In Italy, an average RAP content of 20–30%wt. is usually introduced in WMA and HMA mixes, alternatively [13,14]. The aged and more brittle bituminous binder that coats the recycled aggregates limits the use of RAP material, as it stiffens the final asphalt mixtures, making the pavements more brittle and sometimes more prone to cracking, especially at low temperatures. To overcome this problem, a rejuvenating agent could be used [15,16].

Every year, the European Union (EU) produces around 15 million tons of bitumen [17]. Most of this amount is mixed with aggregates to create asphalt concrete for roads paving. Approximately 90% of all paved roads are surfaced with bituminous materials. Annually, the EU produces more than 200 million tons of bituminous materials for maintenance operations on the existing asphalt pavements and for paving new transportation infrastructure [18]. However, the bitumen used and the virgin aggregates are non-renewable resources. This has led researchers to look for alternative binders (or eco-binders) to reduce the consumption of petroleum bitumen and for recycled aggregates to substitute the virgin ones in the asphalt mixture skeleton. On one hand, the employment of recycled aggregates is becoming a consolidated practice in the production of asphalt concretes, especially in terms of RAP material as previously mentioned, and the use of recycled asphalt mixture with high or very high content of RAP is becoming feasible [19,20]. On the other hand, the partial substitution of neat bitumen with recycled materials and/or by-products is still a challenge. The possible substitutes for neat bitumen can either come from recycled materials from non-renewable resources such as REOBs or come from renewable resources such as wood or vegetable waste oils. The bituminous binders that partially consist of the latter products are referred as bio-binders [21,22], which are an eco-friendly alternative to bitumen obtained from non-petroleum-based renewable resources. The chemical composition of the majority of these alternative binders is similar to that of a traditional bitumen, which includes resins, saturates, aromatics, and asphaltenes [23]. Regardless of the origin of the bitumen substitutes, i.e., renewable or non-renewable resources, the waste and/or recycled products can partially replace neat bitumen. In general, substitutes can be introduced in neat bitumen in four different ways based on the type and quantity of waste materials and/or by-products used, and they are referred to as [24]: (a) Bitumen modifier (<10%wt.); (b) Bitumen fluxes (7–15%wt.); (c) Bitumen extender (25–75%wt.); (d) Alternative binders (>75%wt.). Currently, the bitumen modifiers, fluxes, and extenders are the most-used solutions to add waste materials and/or by-products to bituminous binders for the production of asphalt mixtures. Nonetheless, the use of alternative binders to partially and/or completely replace the bitumen in new asphalt formulations is the final goal of the current research inspired by the circular economy concept. Up to date, several different alternative binders have been studied, including engine oil residue, soybean oil, palm oil, fossil fuel, swine waste, and materials from pyrolysis [25]. Different vegetable oils have been investigated in recent times to determine their physical and chemical properties and to evaluate their applicability as bio-binders in the pavement industry [26,27,28]. Bio-oils are produced from plant matter and residues, such as municipal wastes, agricultural crops, and by-products from agriculture and forestry. Other biomass sources include molasses, rice, sugar, potato starches, corn, gum resins and natural tree resins, vegetable oils, natural latex rubber, cellulose, lignin, palm oil waste, peanut oil waste, coconut waste, potato starch, canola oil waste, dried sewerage effluent, and others [29]. Rauf and Williams [23] have conducted a study about bio-oils. They have produced different bio-oils from different sources, i.e., oakwood, switch grass, and corn stover, which exhibited similar behaviour to neat bitumen. Fini et al. [30] produced bio-oil from swine manure and used it as a partial replacement of bitumen. This recycled product was a promising candidate for partial replacement for standard bitumen. In particular, this bio-binder would improve the low-temperature properties of a petroleum-based binder while reducing asphalt pavement construction costs. A Dutch study tested asphalt roads and cycle paths paved with a bitumen-like product made from the natural binder lignin [31]. Lignin is a structural polymer in plants and trees that is released as a waste product from a number of industrial processes. The used lignin came from various sources including different types of paper pulp production and a bio-refinery that produces cellulosic ethanol from straw. On the demonstration roads, the material appeared to be performing in a similar way to a standard bitumen, and a slight noise reduction was observed. In 2018, Yang et al. developed a process to break down the organic parts of household waste, e.g., food waste, plastic, paper, and textiles, to produce a sticky, gloopy black liquid that is very similar to bitumen [32]. The bio-bitumen was produced by pyrolysis. By changing the processing parameters, such as temperature, processing time, and product collection strategy, the research team was able to alter the characteristics and quantities of the final bitumen-like substance. In conclusion, this preliminary study on alternative binders tried to fulfil circular economy goals by identifying suitable additives, coming from recycled materials and industrial by-products, in order to modify REOBs and to achieve the minimum rheo-mechanical performance required for bituminous binders and mixtures. Thus, the new alternative binders underwent rheological and mechanical analysis to measure some of the parameters required for road construction materials. Nevertheless, beyond this preliminary research, additional investigations are needed in order to fully characterize the rheo-mechanical performances and durability of the binders and the corresponding asphalt mix.

## 2. Materials

Various recycled materials and/or by-products were used to define alternative binders intended to fully replace neat bitumen for the production of more sustainable asphalt mixtures. The main constituents of alternative binders are REOBs that have been modified by specific additives to obtain a material similar to the standard bitumen. 

### 2.1. REOBs

Two different REOBs were supplied by Itelyum Regeneration s.r.l, Lodi (LO), Italy. The by-products were produced in two distinct refinery plants of the same company and are marketed as Viscoflex 1000^®^ (V1) and Viscoflex 2000^®^ (V2TQ). The by-product V2 is usually fluxed with low molecular weight oils—in a quantity equal to 20 or 30%wt.—to facilitate materials handling. In particular, the fluxed REOB with 20%wt. and 30%wt. of the low molecular oil are referred to hereafter as V2F (F stands for fluid) and V2D (D stands for dense), respectively. The two petroleum-based by-products are viscous liquids at room temperature and are obtained by different processes. The V1 is the heavier fraction, obtained in a distillation column working at 365 °C and 15 mmHg; while the V2 is obtained through a propane de-asphalting process. Table 1 reports the main physico-chemical characteristics of both products. 

**Figure 1:**
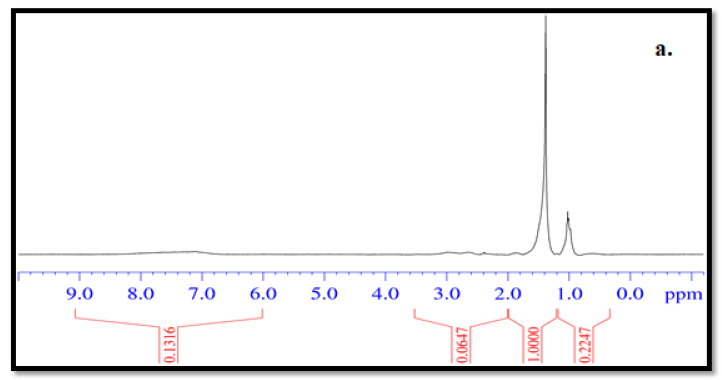

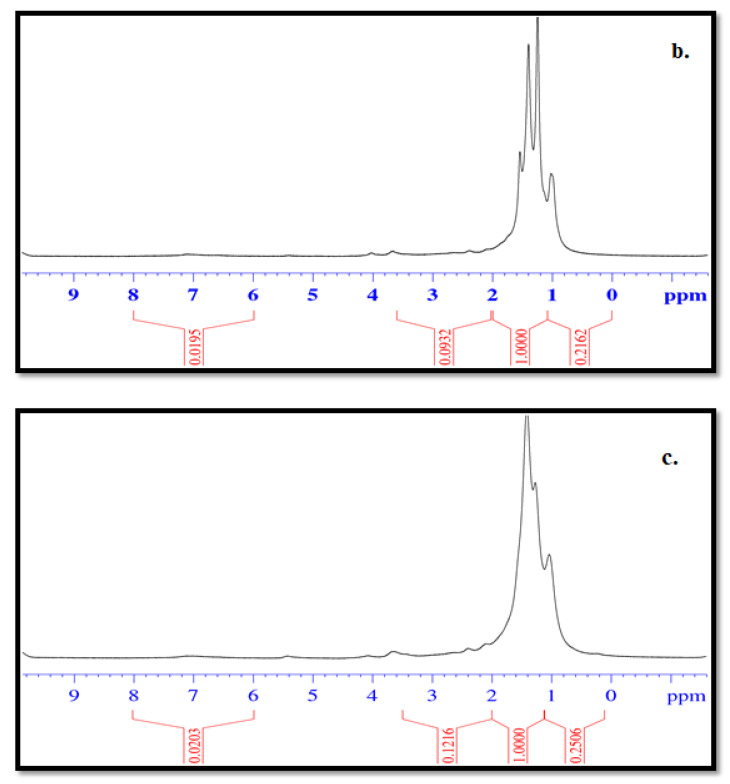

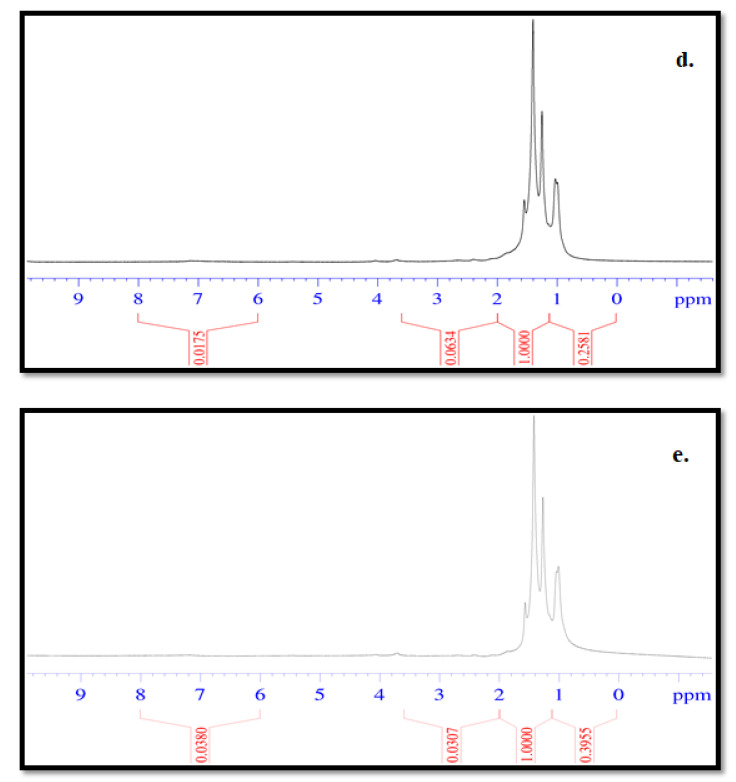
^1^H-NMR spectra of (**a**) Pen 50/70 (**b**) V2TQ, (**c**) V1, (**d**) V2D, (**e**) V2F.

With the aim of replacing neat bitumen mainly with REOBs, the two available REOBs were preliminarily characterized by the use of spectroscopic analysis in order to identify the possible similarities of these materials with a standard 50/70 penetration grade bitumen (Pen 50/70). The Pen 50/70 was considered as the reference bituminous binder throughout the present study. The REOBs were firstly characterized through high-resolution 1H-NMR spectroscopy. In Figure 1, the 1H-NMR spectra of (a) Pen 50/70, (b) V2TQ, (c) V1, (d) V2D, and (e) V2F are reported. As can be seen from Figure 1, all samples are characterized by a rich aliphatic part (0.8 to 2.5 ppm). The areas under the curves for the aliphatic part are of the same order of magnitude for the 50/70 reference bitumen as well as for all REOBs samples. From this point of view, it can be said that REOBs and classic bitumen are very similar., On the other hand, it can be seen from the integral of the aromatic region that the various REOBs have a total aromatic content (asphaltene plus aromatic molecules) about one order of magnitude lower than Pen 50/70. This is in accordance with the results of the analysis shown in Table 1.

### 2.2. Additives

The polymers were introduced in the composition of the alternative binders to improve the elastic response of the final formulations. The powdered rubber from ELTs was supplied by Ecopneus s.c.p.a. The product is a black powder with a maximum size dimension of about 42 μm. Moreover, the introduction of SBS polymer has been considered, since this elastomeric polymer is commonly used in the road sector for the production of modified bitumens. In this study, the amount of SBS used was limited to a maximum of 2% in order to reduce the production costs of the final blends. In addition, it is well-known that SBS polymers work well in combination with rubber from ELTs [12,33]. The SBS polymer was supplied by Kraton Polymers LLC. In order to improve the adhesion of REOBs to the lithic skeleton, an adhesion promoter (AP) has been employed, since it was proven to be effective [34,35]. To modulate the viscosity, various cellulose polysaccharides were used as viscosifier: P2, nano-fibrillated cellulose (CNF), and nanocrystalline cellulose (CNC) [36]. Additionally, to ensure a good workability of the asphalt mix at high temperatures, waxes with melting points of about 100 °C were used, which are commonly used for the production of WMA mixtures [37,38]. In particular, Sasobit waxes (Sb) were employed in this study. Some other issues observed during the blend preparation and analysis have been solved by the aid of other additives such as pine resins (PR) [29,39], which improve the cohesive properties of the REOBs; thickening agents such as Lithium salt (LiS) and nanotubes (Nt) have been used to take advantages of their gelling properties (widely employed in industrial grease production). The PR, AP, and P2 were supplied by Kimical s.r.l. The lithium salt (LiS) was supplied by Sigma Aldrich, while the nanotubes (Nt) were produced in laboratories of the University of Calabria. The nano-fibrillated cellulose (CNF) was supplied by Nanografi Co. Inc., while nano-crystalline cellulose (CNC) was obtained in the laboratories of the University of Calabria from waste papers following the methods reported in the reference [40]. All the supplied products were used without any further treatment. The mean cost of the blends, considering the various additives’ prices and percentages used in our blend preparation, is about 350–400 €/ton.

### 2.3. Alternative Binders

The additives that were used allowed for the definition of final alternative binders similar to the traditional bitumen used for paving. In total, 18 alternative binders were defined and then characterized at the binder and the asphalt mixture levels, which are listed in Table 2. Each alternative binder description consists of all materials that were used in quantity greater than 0%. Hence, each line of Table 2 represents the recipe of one alternative binder.

Prior to samples preparation, the various additives underwent thermogravimetric analysis in order to check their stability at the high temperature, i.e., 160 °C. No additives showed considerable weight loss and, consequently, no additives degradation would occur during mixing process. 

The preparation of alternative binders required the preliminary heating phase of each specific REOB (V1, V2TQ, V2F and V2D) at about 160 ± 5 °C. Then, the quantity of the chosen additives was gradually added to the warmed REOB (1 g/min). The additives were incorporated at room temperature. All constituents were mixed by means of high-shear mixer (IKA model) with an average speed of about 1400–1600 rpm. Each blend was mixed at 160 °C for 60 min to guarantee an essentially homogenous sample.

### 2.4. Asphalt Mixtures

The asphalt mixes with the alternative binders were produced using RAP aggregates only. The recycled aggregates consisted of milled asphalt concrete from existing pavements of highways. The grading distribution and the binder content were designed in order to produce a traditional wearing course asphalt mixture. The grading distribution of the RAP aggregates met the Italian technical specifications. Based on the aged bituminous binder content already present in the recycled aggregates and on previous studies based on Cantabro test (EN 12697-17) [41], the optimum binder content was selected as equal to 2.5% of the total weight of aggregates. The innovative asphalt mixtures were compared with samples of a traditional wearing course layer made of 90% virgin aggregates and 10% RAP and Pen 50/70, which was considered as a reference mix, and a mix consisting of 100% RAP aggregate and Pen 50/70. Moreover, the limits of the Italian technical specifications for wearing course layers were considered as reference for comparisons.

All alternative binders were used for producing 18 different asphalt mixtures. Per each asphalt concrete mix, three samples were manufactured. The cylindrical samples had diameter of 100 mm and were 55 mm tall, approximately. The correct amount of recycled aggregates and the alternative binders were preliminarily heated in an air-forced oven before being mixed and compacted. Per each asphalt mix, 3000 g of RAP aggregates were heated at 150 °C for 2 h and 75 g of the prepared blends (i.e., 2.5%wt.) at 150 °C for a minimum of 1 h. The samples underwent compaction by means of a gyratory compactor applying 100 gyrations at 600 kPa [42]. 

## 3. Test Methods

In order to preliminarily assess the feasibility of using alternative binders for road construction materials, the 18 new binders underwent rheological analysis. Then, the mechanical response of all asphalt mixtures, those containing an alternative binder and the reference mixes, were investigated. Since these were binders without bitumen as the main constituent, the basic tests were planned with the aim of assessing a possible relationship, if any, between the behaviour of binders and that of mixtures as they exist for traditional bituminous materials.

### 3.1. Rheological Measurements

A dynamic shear rheometer (SR5000, Rheometric Scientific, Piscataway, NY, USA) was used to perform the rheological tests on the various alternative binders. The controlled shear stress rheometer was used in a plate–plate configuration. Plate tools of ϕ = 25 mm diameter were used for testing in the temperature range of 25–120 °C. The gap was set equal to 2 mm. A Peltier system (±0.1 °C) controlled the test temperature. The rheological responses of Pen 50/70 and alternative binders were determined under the kinematics of both steady and oscillatory simple shears. In steady-shear experiments, the viscosity of blend samples was determined from the ratio of measured shear stress to applied shear rate, as a function of shear rate that varied from 1 to 100 s^−1^. Steady states were previously checked by transient experiments (step-rate test). For all samples, it was observed that 10 s was a sufficient scanning time to ensure the steady-state condition. All samples showed a Newtonian behaviour in the investigated shear rate range. Dynamic tests were carried out in conditions of linear viscoelastic (LVE) region, where measured material features do not depend on the amplitude of applied load and are related to materials microstructure only. With the aim of investigating the material viscoelastic phase transition, dynamic temperature ramp tests (DTRT) were performed both at 1 Hz and temperature rate of 1 °C/min from 25 °C to 120 °C by applying the proper stress values—previously determined by stress sweep tests—to guarantee linear viscoelastic conditions at all tested temperatures. 

### 3.2. Mechanical Analysis

The resulting 18 asphalt mixtures were subjected to dynamic and static mechanical characterizations. The asphalt concrete samples underwent mechanical tests after being cured for a minimum of 24 h. Dynamic tests were used to determine the Indirect Tensile Stiffness Modulus (ITSM) at 20 °C of all samples by using a servo-pneumatic testing machine. The ITSM values were determined according to EN 12697-26 standard [43], in the indirect tensile configuration (IT-CY). A pulse loading was applied with a 124 ms rise-time to generate a horizontal deformation of 5 ± 2 μm. Two static mechanical characterizations were used to measure the Indirect Tensile Strength (ITS) and the Indirect Tensile Strength Ratio (ITSR) of all mixes according to the EN 12697-23 [44] and EN 12697-12 [45] standards, respectively. The tensile resistance of asphalt concretes was determined by applying a compression load with a constant speed rate of 51 mm/min. The ITS test was performed at 25 °C. The latest characterization, i.e., the ITSR ratio, aimed to assess the durability of the wearing course samples, as it determines the effect of water conditioning. This investigation quantifies the ratio between the ITS values of an asphalt mix after water conditioning and those of dry specimen. According to standard method A, the samples were saturated while stored in a water bath at 40 °C for three days. Successively, the samples were removed, dried, and conditioned at 25 °C in a climate chamber to further undergo ITS testing. The two static characterizations applied a load until failure; hence, two samples were used to evaluate the average ITS values, while the third specimen of each asphalt mix was used to determine the corresponding ITSR value. Before being tested, all samples were kept in a climate chamber at the test temperature for at least 4 h.

## 4. Results and Discussion

### 4.1. Rheological Analysis

**Figure 2:**
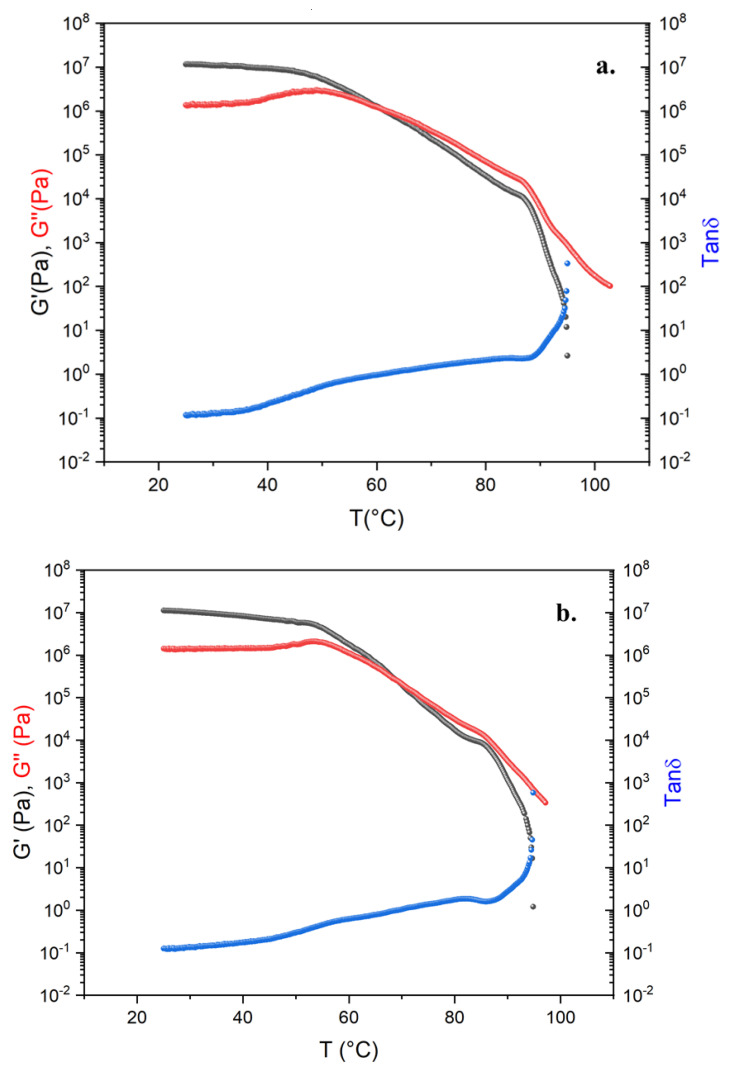

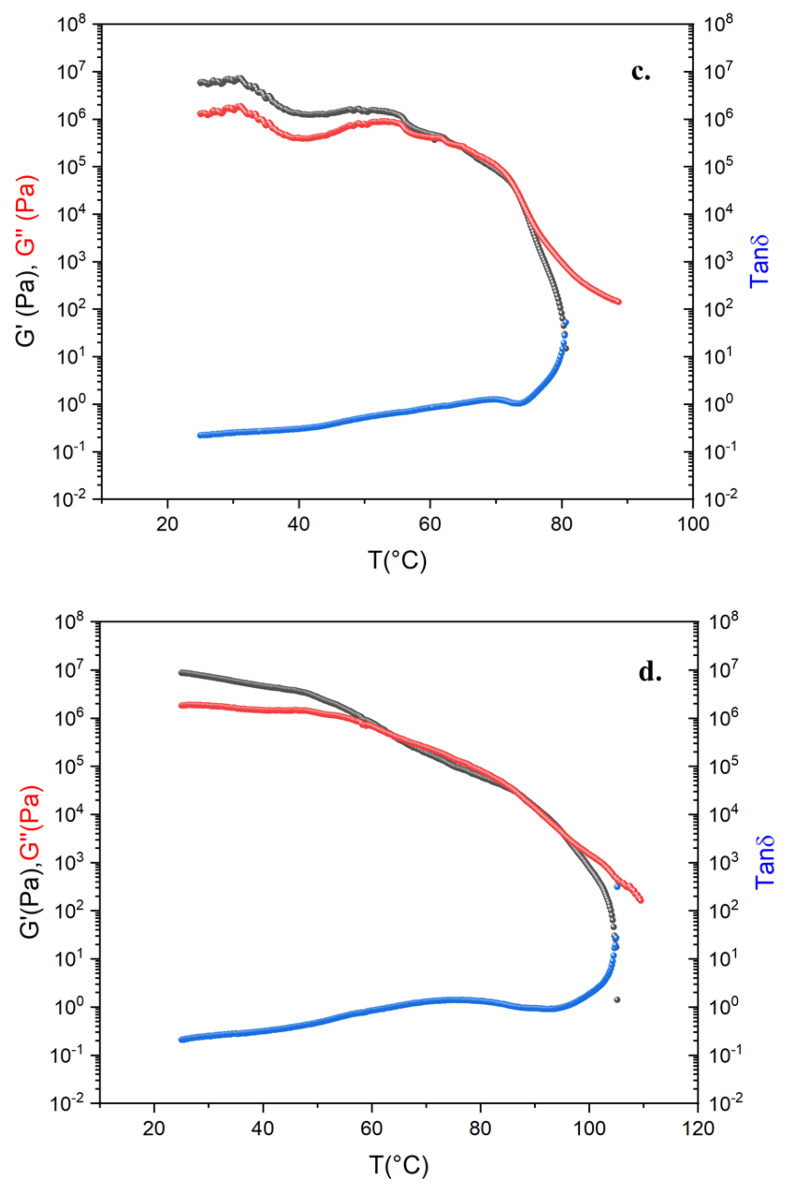
Dynamic Temperature Ramp Test (DTRT) of (**a**) blend 26-1, (**b**) blend 29-1, (**c**) blend 27-1, (**d**) blend 32V1.

**Figure 3:**
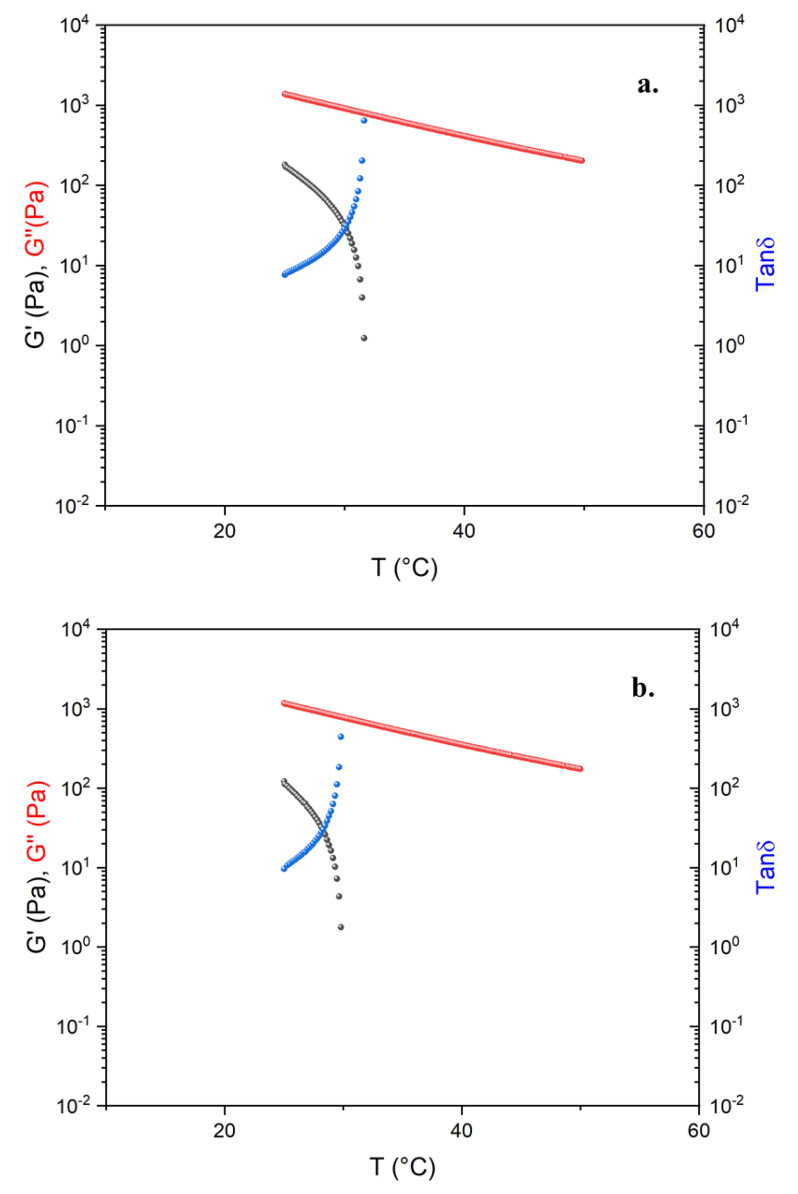

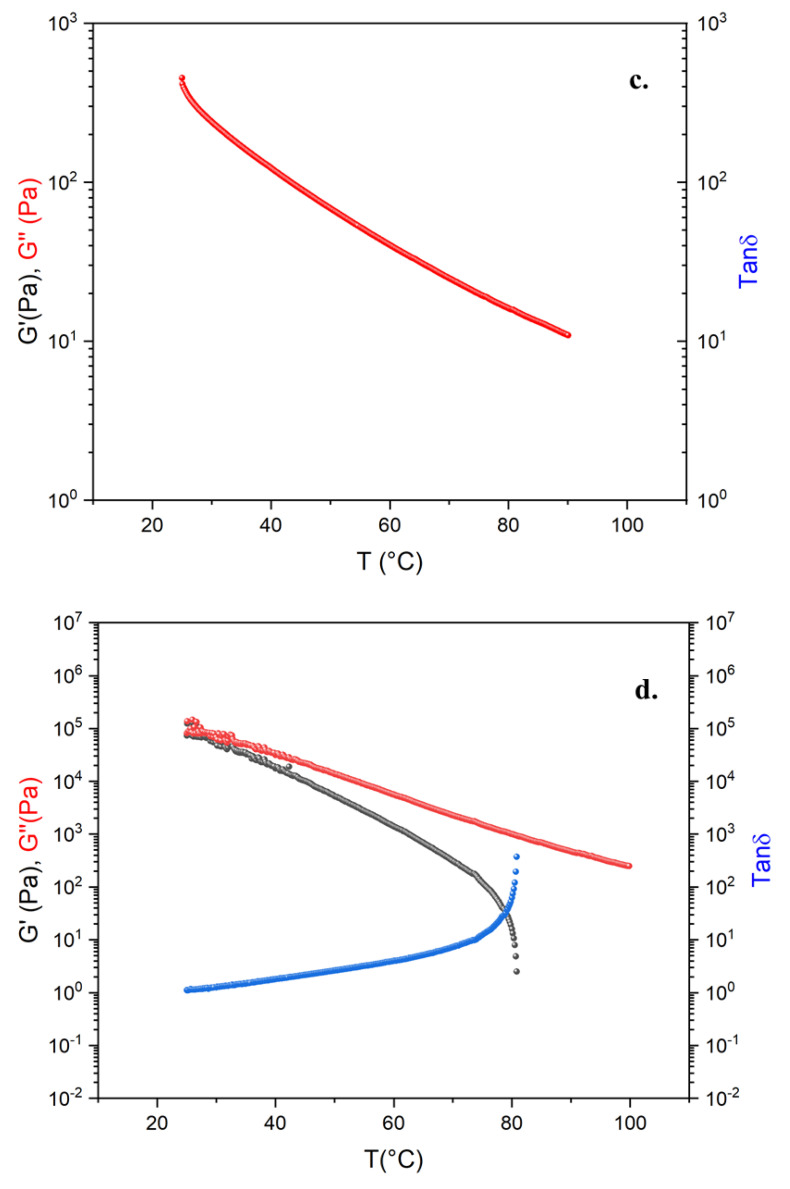

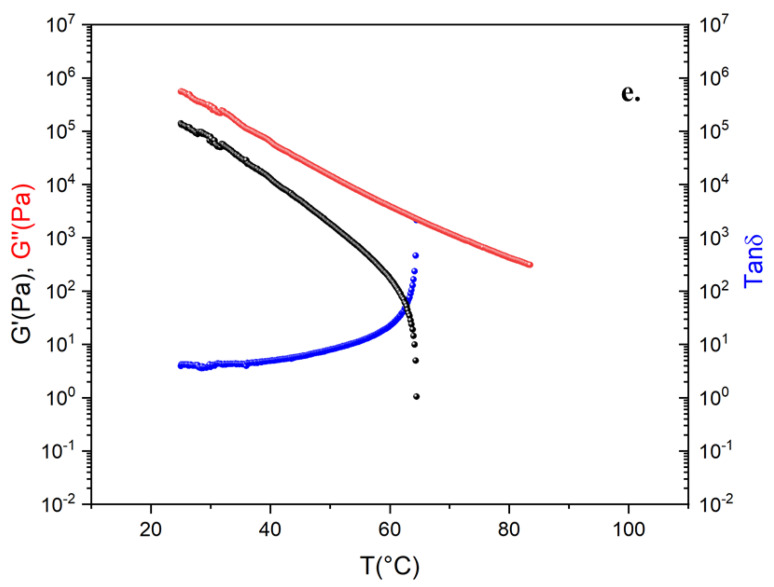
Dynamic Temperature Ramp Test of (**a**) V2F, (**b**) V2D, (**c**) V1, (**d**) V2TQ, (**e**) Pen 50/70.

**Figure 4:**
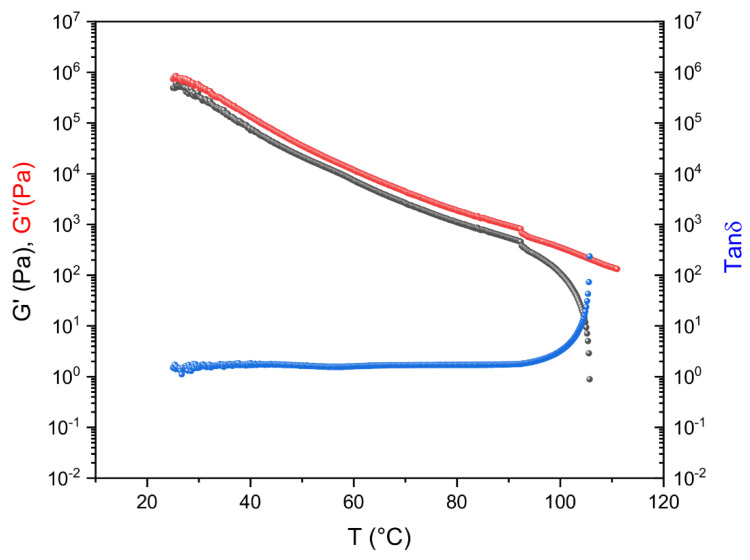
Dynamic Temperature Ramp Test of an SBS-modified bitumen (Pen 50/70 + 3% SBS).

Figure 2 shows the results of the DTRT obtained from the blends B26-1, B27-1, B29-1, and B32V1. Among all alternative binders, these four blends were selected because they exhibit good rheological and mechanical responses, as can be seen by comparing the DTRT of our blends with that of a 50/70 bitumen reported in Figure 3. The remaining blends that were prepared show good rheological behaviour, but they do not behave as expected from a mechanical point of view (the DTRT of the other blends can be found in the Supplementary Information Figure S1). The DTRT of virgin REOBs (V2F, V1, V2D, V2TQ) are reported in Figure 3. Figure 2 and Figure 3 shows that alternative binders strongly enhance the rheological properties of both REOBs and bitumen. Moreover, by comparing the DTRT of the alternative binders—even those reported in the Supplementary Information—with that of an SBS-modified bitumen (PmB) reported in Figure 4, it can be concluded that the alternative binders resemble the behaviour of a polymer-modified bitumen. 

### 4.2. Mechanical Analysis

**Figure 5:**
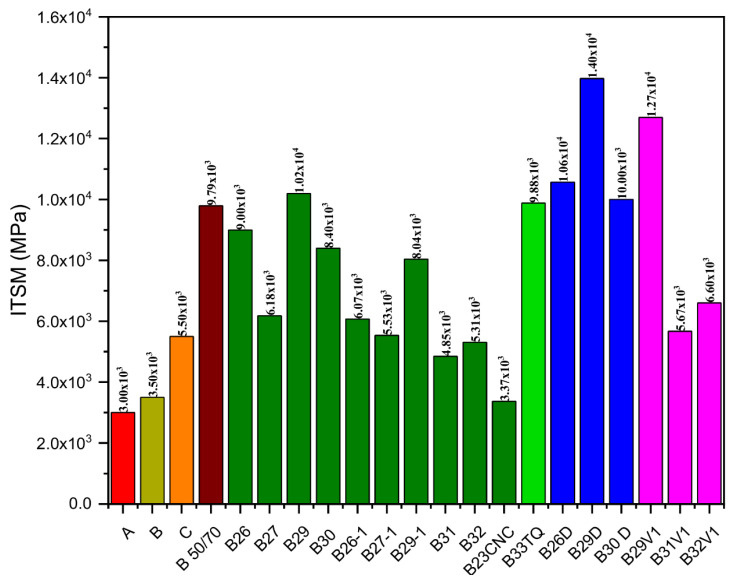
Results of the ITSM values at 20 °C for the recycled asphalt mixtures produced with 100% RAP and the alternative binders. Legend: 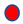 Asphalt concrete with virgin bitumen and aggregates (A); 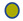 Asphalt concrete with virgin polymer-modified bitumen and aggregates (B); 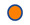 Reference mix (C); 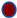 100% RAP asphalt concrete with Pen 50/70; 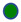 100% RAP asphalt concrete with alternative binders containing V2F; 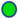 100% RAP asphalt concrete with B33TQ; 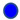 100% RAP asphalt concrete with alternative binders containing V2D; 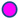 100% RAP asphalt concrete with alternative binders containing V1.

The dynamic mechanical characterization of the asphalt mixtures manufactured with the 18 alternative binders and Pen 50/70 is reported in Figure 5. The asphalt mixes exhibit high variability in the ITSM results, which can be ascribed to the constituent materials used. The mixture with B23CNC shows the lowest stiffness modulus, while the mixture containing B29D has the highest stiffness. In general, the asphalt mixes containing the V2D show higher stiffness than the corresponding asphalt concrete mixtures with V2F. The higher presence of low-molecular-weight oil in the alternative binders lead to soften the final materials as expected. Hence, the type of REOB affects the final ITSM values. Additionally, the combination of specific additives permits alternatively increasing or decreasing the stiffness modulus of the corresponding asphalt mixture. In fact, the B33TQ mix has lower ITSM value than the B29D asphalt concrete. In general, the minimum ITSM values at 20 °C of a traditional wearing course layer made with virgin materials only are 3000 MPa and 3500 MPa for samples produced with unmodified and polymer-modified bitumens, respectively. Previous studies on the reference mix that contains 10% of RAP and Pen 50/70 have assessed an average ITSM value equal to 5500 MPa at 20 °C, which can be used for comparing the stiffness modulus of the innovative asphalt mixtures with the alternative binders. Among all tested asphalt materials, the mixtures manufactured with B26-1, B27, B27-1, B31, B31V1, B32, and B32V1 have similar stiffness to the reference mixture that mainly contains virgin materials. Most of the recycled asphalt mixtures with alternative binders have higher stiffness than the samples of the reference mix, which can be mainly related to the presence of RAP and to the addition of PR. In general, the use of recycled aggregates has been found to stiffen the final asphalt concretes; indeed, the mixture with 100% RAP and Pen 50/70 is stiffer than the reference one. The introduction of PR in the bituminous binders improve the cohesion properties, which turn in higher ITSM values. Indeed, the alternative binders with a high content of PR are very stiff. However, the use of asphalt mixtures with a very high stiffness is disadvantageous, as the resulting asphalt pavement may be more prone to fatigue and thermal cracking.

**Figure 6:**
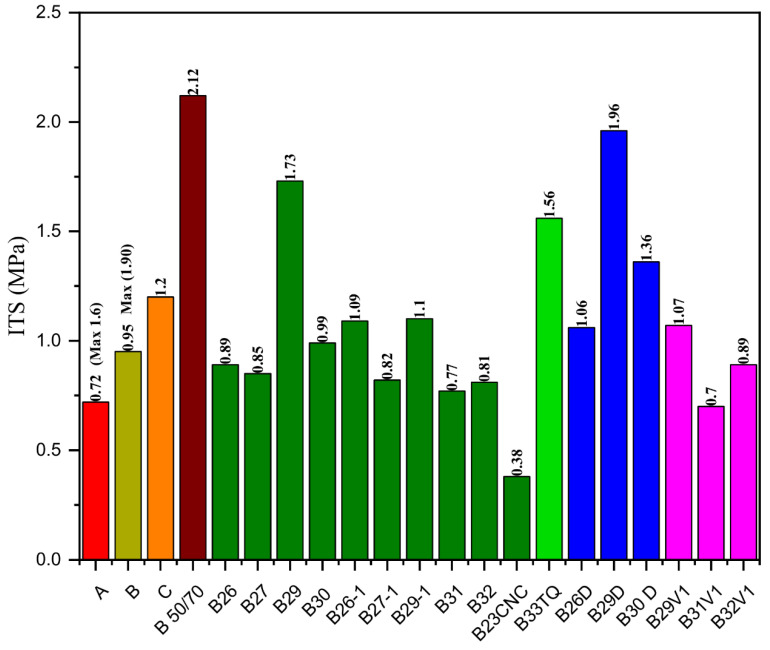
Results of the ITS values at 20 °C for the recycled asphalt mixtures produced with 100% RAP and the alternative binders. Legend: 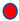 Asphalt concrete with virgin bitumen and aggregates (A); 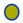 Asphalt concrete with virgin polymer-modified bitumen and aggregates (B); 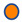 Reference mix (C); 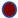 100% RAP asphalt concrete with Pen 50/70; 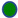 100% RAP asphalt concrete with alternative binders containing V2F; 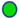 100% RAP asphalt concrete with B33TQ; 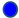 100% RAP asphalt concrete with alternative binders containing V2D; 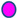 100% RAP asphalt concrete with alternative binders containing V1.

Figure 6 shows the indirect tensile resistance of the innovative asphalt mixtures produced with RAP and alternative binders. The Italian technical specifications require a minimum ITS value equal to 0.72 or 0.95 MPa for asphalt concrete with unmodified and polymer-modified bitumens, respectively. Almost all innovative mixtures meet the requirement; only the innovative mixtures with B23CNC, B27-1, and B29-1 show insufficient tensile resistance. The recycled mix with Pen 50/70 exhibits the highest tensile resistance. For bituminous materials, the tensile resistance and stiffness values are directly related to each other, and this correlation is also confirmed in the present study. The innovative asphalt mixes with the highest ITSM values exhibit the highest ITS results. However, the ITS values close to or higher than 2.0 MPa may reflect a very high stiffness, which may turn into brittle asphalt pavements. As a consequence, the Italian technical specifications limit the tensile resistance of the asphalt mixes, and the ITS values of the asphalt mixtures with neat and polymer-modified bitumen have to be lower than 1.60 and 1.90 MPa, respectively. In this regard, the samples manufactured with B26, B26D, B26-1, B27, B27-1, B29V1, B29-1, B30, B30D, B31, B32, and B32V1 meet the required specifications for mixtures with unmodified bituminous binders. These innovative mixes have a similar response to the reference mix with virgin materials, as their ITS values are equal to 1.2 MPa on average.

**Figure 7:**
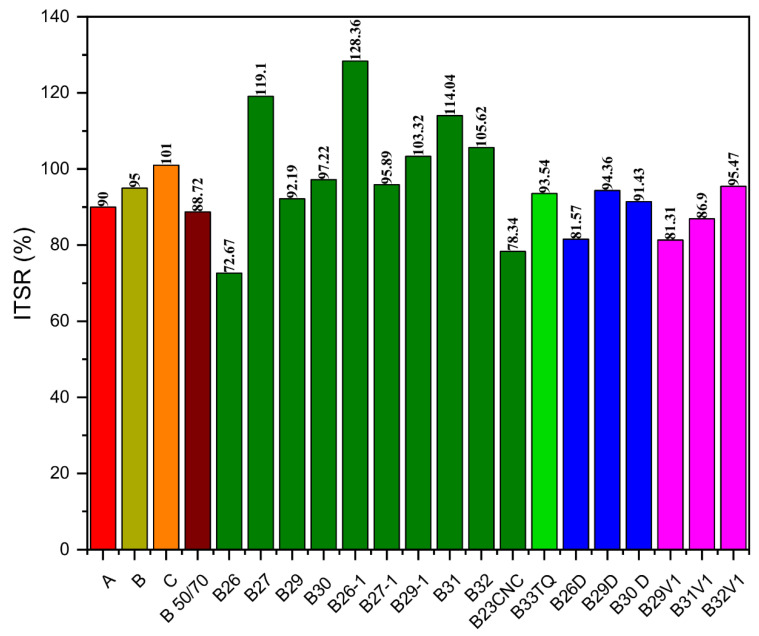
Results of the ITSR values at 20 °C of the recycled asphalt mixtures produced with 100% RAP and the alternative binders. Legend: 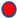 Asphalt concrete with virgin bitumen and aggregates (A); 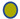 Asphalt concrete with virgin polymer-modified bitumen and aggregates (B); 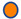 Reference mix (C); 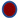 100% RAP asphalt concrete with Pen 50/70; 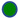 100% RAP asphalt concrete with alternative binders containing V2F; 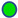 100% RAP asphalt concrete with B33TQ; 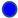 100% RAP asphalt concrete with alternative binders containing V2D; 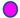 100% RAP asphalt concrete with alternative binders containing V1.

The results of measuring the water susceptibility for the innovative asphalt mixtures are reported in Figure 7. The type of REOB significantly affect the resistance of asphalt concrete, as the use of V1 decreases the water damage resistance of the mixes when compared to the corresponding mixtures with one of the V2s. The minimum ITSR value required by the Italian technical specifications is about 90% when unmodified bitumens are used. Among the innovative asphalt mixtures, almost all mixes that contain V2F (except for B26 and B23CNC) and the mixtures made with B33TQ, B29D, B30D, and B32V1 show good resistance against water damage, or they do not even show any water susceptibility, as the ITSR results are greater than 100%. This behaviour can be ascribed to the presence of a very high quantity of RAP aggregates. The abovementioned asphalt concrete mixes behave similarly to the reference mix mainly produced with virgin aggregates. On the other hand, the ITS value of the 100% RAP mix with Pen 50/70 is considerably reduced after water conditioning, and its ITSR ratio is about 89%.

Among all analysed asphalt mixtures, those containing the blends B26-1, B27, B27-1, B31, B32, and B32 V1 exhibit good mechanical properties in terms of stiffness, tensile resistance, and water susceptibility. Although the results obtained with the alternative binders are promising, further specific rheological and mechanical tests have to be carried out in order to assess the feasibility and the durability of these road materials that do not contain standard bitumen. For instance, testing fatigue (mechanical), ageing susceptibility (rheological and mechanical), and low-temperature behaviour of the final alternative binders and asphalt mix will be crucial to establish the performances of these innovative asphalt products. In particular, the ageing tendency of the alternative binders, and consequently of asphalt concrete, has to be evaluated, since an aged material is used, i.e., REOB. Some of the abovementioned tests are ongoing and are showing promising results.

## 5. Conclusions

In this work, the potential reuse of waste products that were opportunely treated to produce new possible petroleum-based binders starting from REOBs is proposed. In addition, the use of 100% recycled aggregates (RAP) together with alternative binders can represent a good alternative to the current use of virgin materials at the binder and asphalt mixture levels. During this study, rheological and mechanical tests were carried out to preliminarily assess the mechanical properties of these innovative binders. From a rheological point of view, the alternative binders exhibit similar behaviour to polymer-modified bitumen (3%wt. of SBS). The type of REOB, the chosen additives, and the combination of constituent materials are found to be crucial to the final responses of the innovative petroleum-based binders and asphalt mixes. In particular, the introduction of low-molecular-weight oils by REOB products softens the resulting alternative binders and, consequently, the asphalt mixtures, resulting in lower ITSM and ITS values. In addition, the use of recycled mixture with 100% of RAP and alternative binders confers good water damage resistance to the corresponding asphalt mix. In general, various eco-friendly asphalt mixtures show promising results in terms of stiffness, tensile resistance, and water susceptibility. In detail, among all tested materials, the asphalt concretes that contained the alternative binders B26-1, B27, B27-1, B31, B32, and B32V1 meet the requirements of the Italian technical specifications for wearing course samples produced with virgin materials. Hence, these innovative mixtures satisfy the basic mechanical performances. However, limited recipe adjustment may allow the stiffness reduction of the abovementioned asphalt mixtures without compromising the cohesion and water sensitivity of the final road materials. Even though the results of the present study are promising, they represent a preliminary evaluation of the performances of the alternative binders, and further rheological and mechanical analyses are necessary. In particular, the durability of the asphalt concrete has to be investigated, considering the natural ageing process of petroleum-based product and the use of an aged material for the production of the alternative binders (i.e., REOB). The determination of eco-friendly road materials is still ongoing, and the responses of the low-temperature DTRT and fatigue tests are under investigation.

## Figures and Tables

**Table 1:** Main physical and chemical characteristics of V1 and V2F.

Property	Standard	Results	
		V1	V2F
Needle Penetration Test	EN 1462	/	>500 (0.1 mm)
Softening Point	UNI EN 1427	/	<4 °C
Density at 15 °C	ASTM D70	1003 kg/m^3^	$0.975\;\mathrm{kg}/m^{3}$
Kinematic Viscosity at 100 °C	ISO 3104	Not determinable	$580\;{\mathrm{mm}}^{2}/s$
Kinematic Viscosity at 135 °C	ISO 3104	$110.0\;{\mathrm{mm}}^{2}/s$	/
Dynamic Viscosity at 60 °C	EN 13702	25.13 Pa s	0.380 Pa s
Pour Point	ASTM D97	>50 °C	/
Insoluble Matter	ASTM D 2042	14% w/w	/
Soluble Matter	ASTM D 2042	86.10% w/w	/
Water Content	ASTM D6304 (Procedure C)	290 mg/kg	/
Sulphur	ISO 8754	1.13% w/w	/
Nitrogen Content	ASTM D3228	0.32% w/w	/
Gasoline Fuel	ASTM D3525	<0.01% w/w	/
Gasoline Diluent	ASTM D322	<0.1% v/v	/
Diesel Fuel	ASTM D3524	<0.1% w/w	/
Ash	ASTM D482	8.089% w/w	/
Conradson Carbon Residue	ASTM D189	19.2% w/w	/
TSE Remark	ISO 10307-1	Filtration Time exceeds 25 min	/
Saturates	IP 469	32.9% w/w	37.0% w/w
Aromatics		0% w/w	1.6% w/w
Polars (I)		19.6% w/w	18.1% w/w
Polars (II)		47.5% w/w	43.3% w/w
Asphaltene	IP 143	16.6% w/w	3.7% w/w
Pensky–Martens Flash Point (Closed Cup) Procedure B	ASTM D93/IP34/EN ISO 2719	270 °C	/
Cleveland Flash Point (Open Cup)	ASTM D92/EN ISO 2592	284 °C	180 °C
PCB Content	EN 12766-3	<4 mg /kg	/
PCT	EN 12766-3	<10 mg /kg	/

**Table 2:** Type and percentage of constituent materials of the 18 alternative binders.

Blend	V1	V2TQ	V2F	V2D	PFU	Sb	SBS	AP	P2	CNC	CNF	PR	Nt	LiS
B23 CNC	-	-	60	-	15	10	-	0.3	-	14.7	-	-	-	-
B26	-	-	40	-	10	10	-	-	20	-	-	40	-	-
B27	-	-	60	-	5	10	-	-	15	-	-	10	-	-
B29	-	-	40	-	10	10	-	-	-	-	-	40	-	-
B29V1	40	-	-	-	10	10	-	-	-	-	-	40	-	-
B30	-	-	40	-	-	8	-	-	14.9	-	-	27	0.1	10
B26D	-	-	-	40	10	10	-	-	20	-	-	20	-	-
B27D	-	-	-	60	5	10	-	-	15	-	-	10	-	-
B29D	-	-	-	40	10	10	-	-	-	-	-	40	-	-
B30D	-	-	-	40	-	8	-	-	14.9	-	-	27	0.1	10
B26-1	-	-	60	-	10	10	1	0.3	8.7	-	-	10	-	-
B27-1	-	-	60	-	5	10	1	0.3	13.7	-	-	10	-	-
B29-1	-	-	50	-	10	10	0.5	0.3	-	-	-	29.2	-	-
B31	-	-	60	-	15	10	2	0.3	7.7	-	-	5	-	-
B31V1	60	-	-	-	15	10	2	0.3	7.7	-	-	5	-	-
B32	-	-	60	-	15	5	-	0.3	-	-	14.7	5	-	-
B32V1	60	-	-	-	15	5	-	0.3	-	-	14.7	5	-	-
B33TQ	-	60	-	-	19.7	13	2	0.3	-	-	-	5	-	-

## Data Availability

The data presented in this study is available upon request from the corresponding author.

## Supplementary Materials

The following are available online. Figure S1: Dynamic Temperature Ramp Test of B26 V2000F; Figure S2: Dynamic Temperature Ramp Test of B26 V2000D; Figure S3: Dynamic Temperature Ramp Test of B27 V2000D; Figure S4: Dynamic Temperature Ramp Test of B27 V2000F; Figure S5: Dynamic Temperature Ramp Test of B29 V1000; Figure S6: Dynamic Temperature Ramp Test of B29 V2000D; Figure S7: Dynamic Temperature Ramp Test of B29 V2000F; Figure S8: Dynamic Temperature Ramp Test of B30 V2000D; Figure S9: Temperature Ramp Test of B30 V2000F; Figure S10: Dynamic Temperature Ramp Test of B31 V2000F; Figure S11: Dynamic Temperature Ramp Test of B31 V1000; Figure S12: Dynamic Temperature Ramp Test of B32; Figure S13: Dynamic Temperature Ramp Test of B33.

## Sample Availability

Samples of the compounds are not available from the authors.
