# Supplementary material for: Preliminary Study on New Alternative Binders through Re-Refined Engine Oil Bottoms (REOBs) and Industrial By-Product Additives
Source: Molecules. 2021 Nov 30;26(23):7269. doi: 10.3390/molecules26237269 (PMC8659085; doi:10.3390/molecules26237269)
Supplement: Supplementary file 1 [file molecules-26-07269-s001.zip › molecules-1461542-supplementary.pdf]

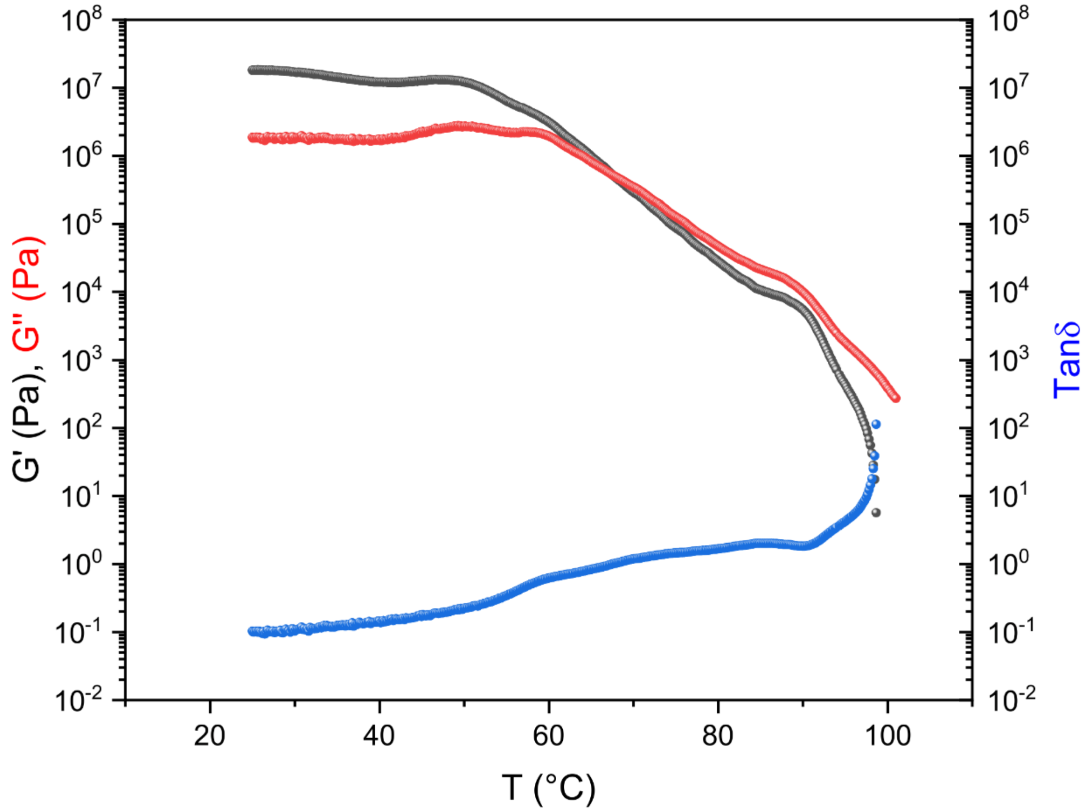

Figure S1 Dynamic Temperature Ramp Test of B26 V2000F.

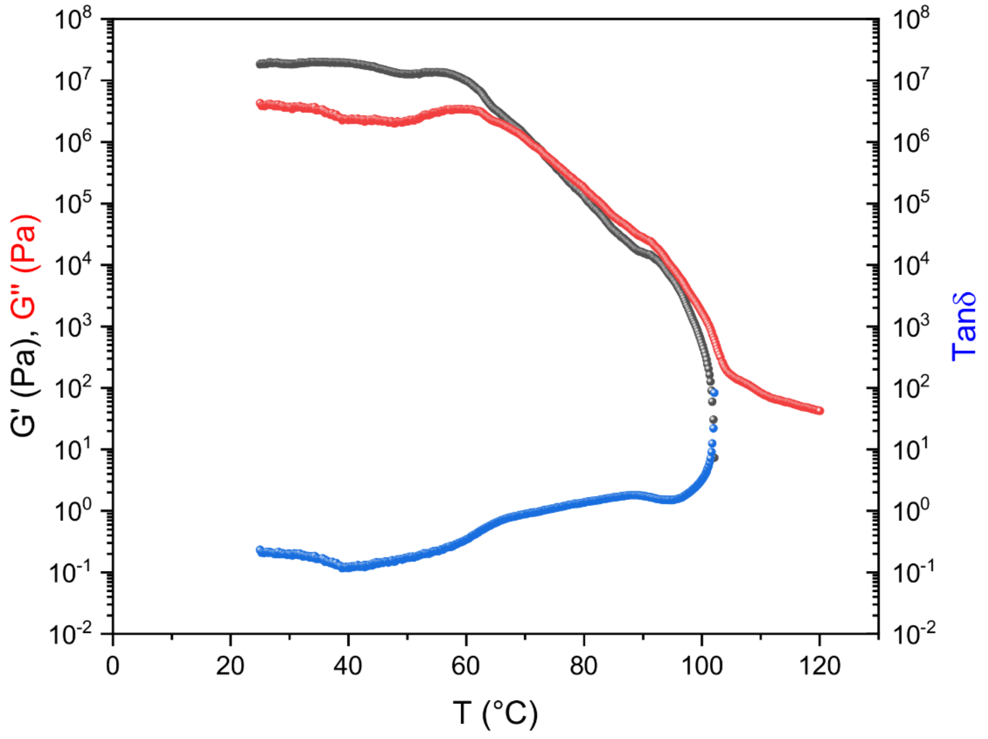

Figure S2 Dynamic Temperature Ramp Test of B26 V2000D.

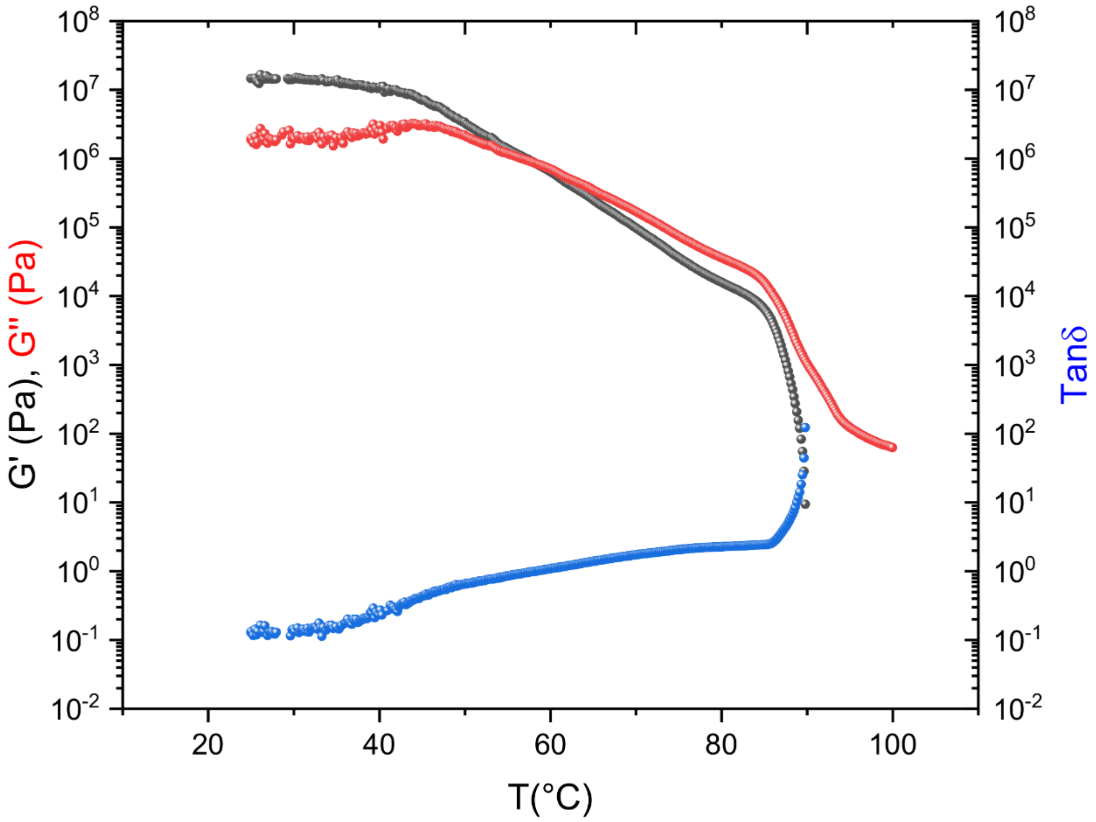

Figure S3 Dynamic Temperature Ramp Test of B27 V2000D.

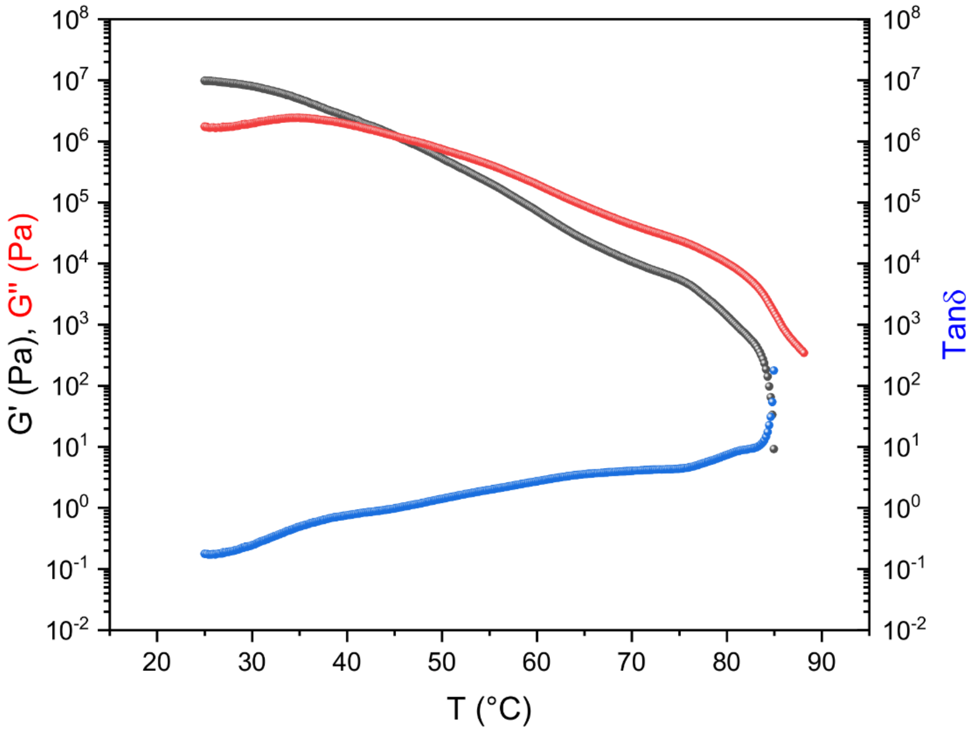

Figure S4 Dynamic Temperature Ramp Test of B27 V2000F.

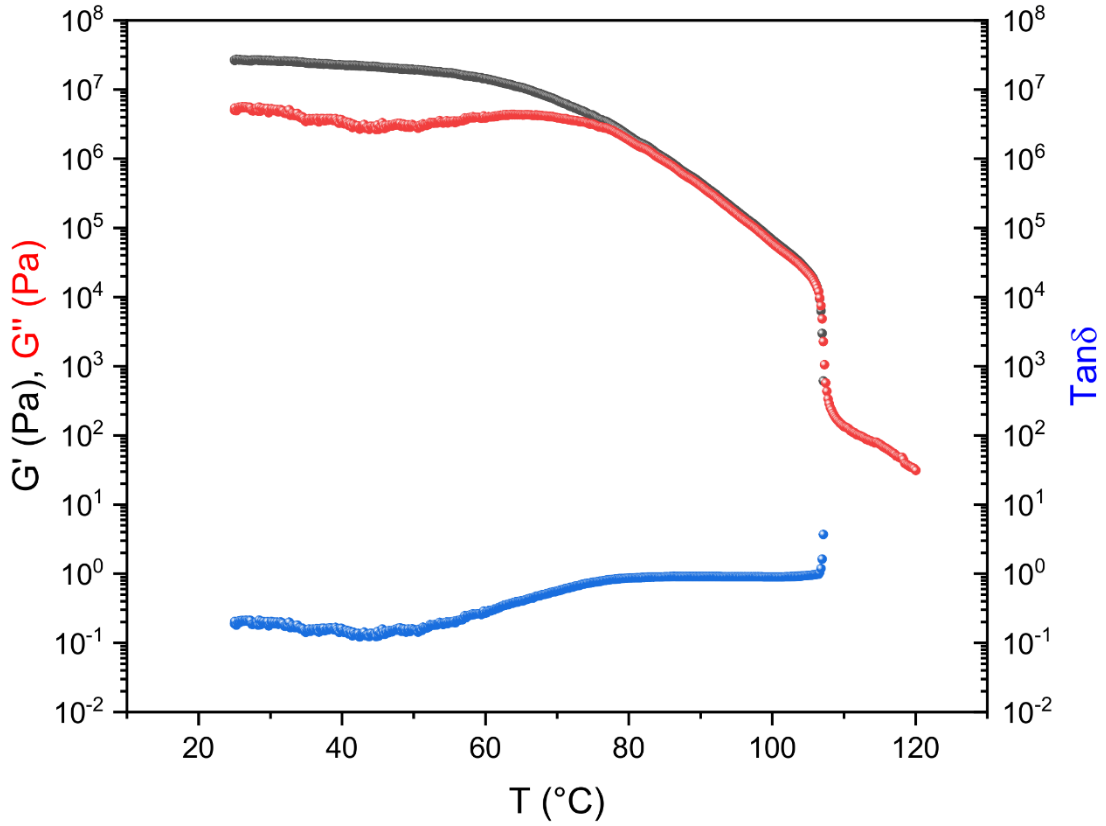

Figure S5 Dynamic Temperature Ramp Test of B29 V1000.

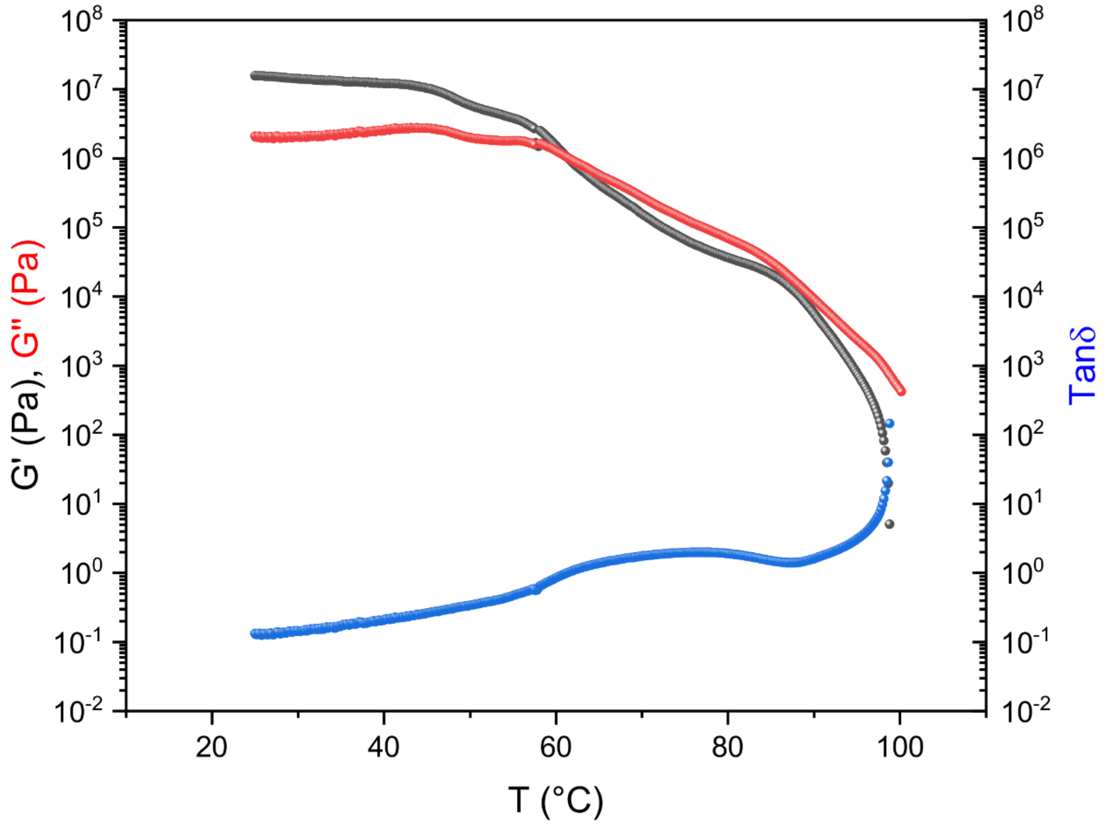

Figure S6 Dynamic Temperature Ramp Test of B29 V2000D.

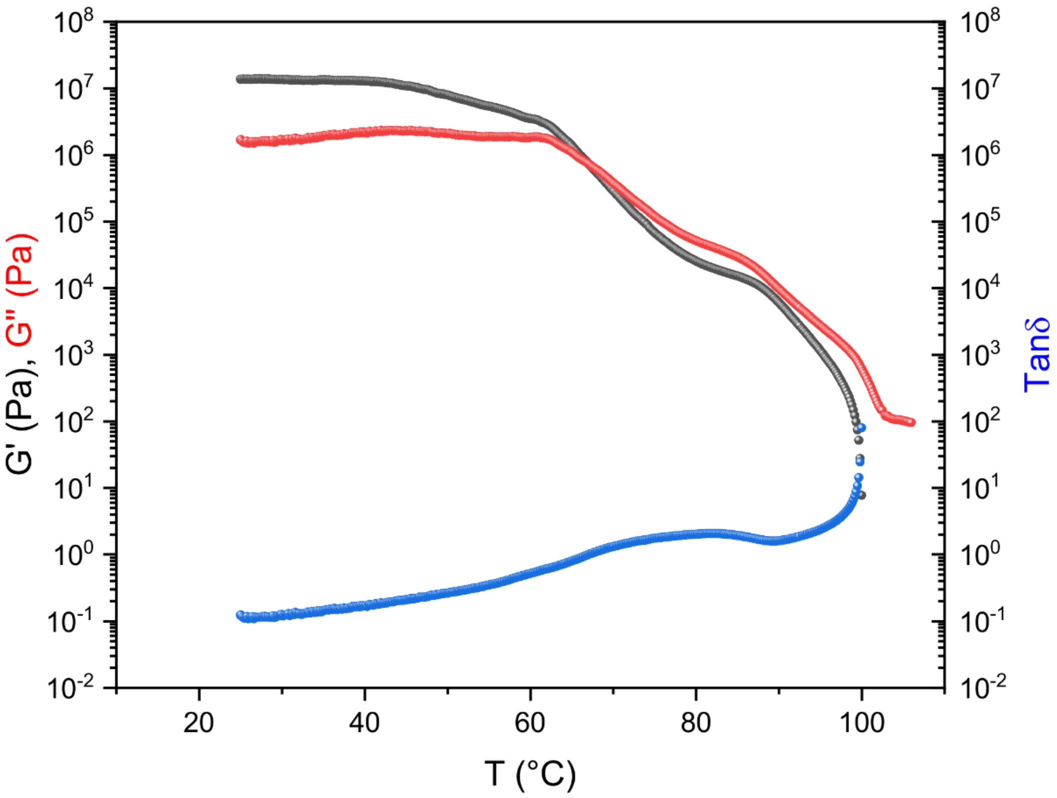

Figure S7 Dynamic Temperature Ramp Test of B29 V2000F.

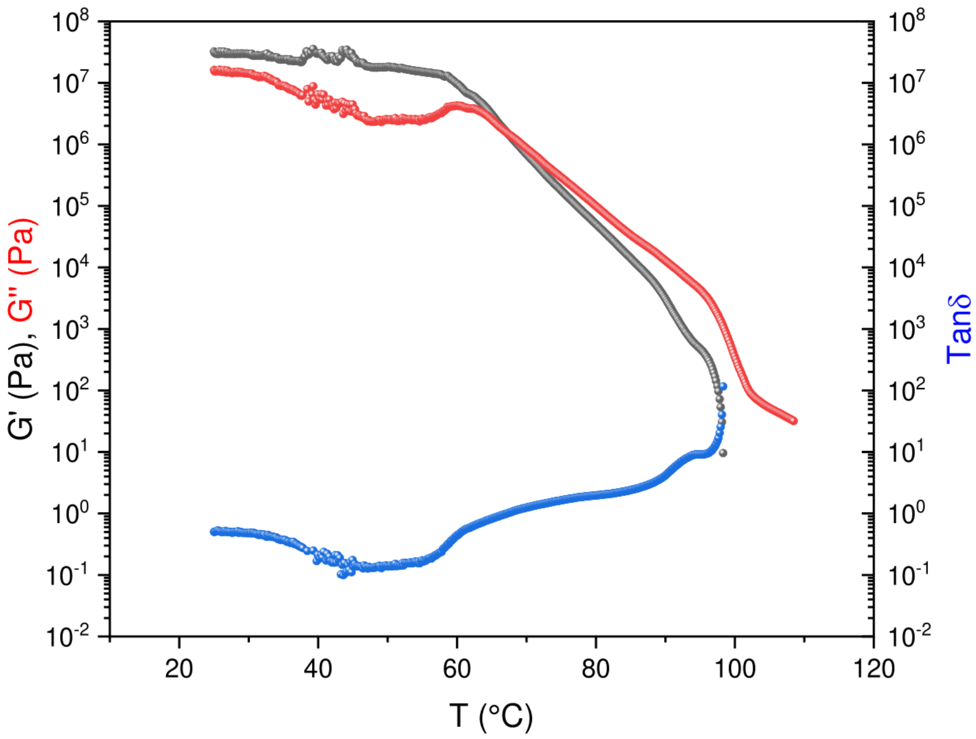

Figure S8 Dynamic Temperature Ramp Test of B30 V2000D.

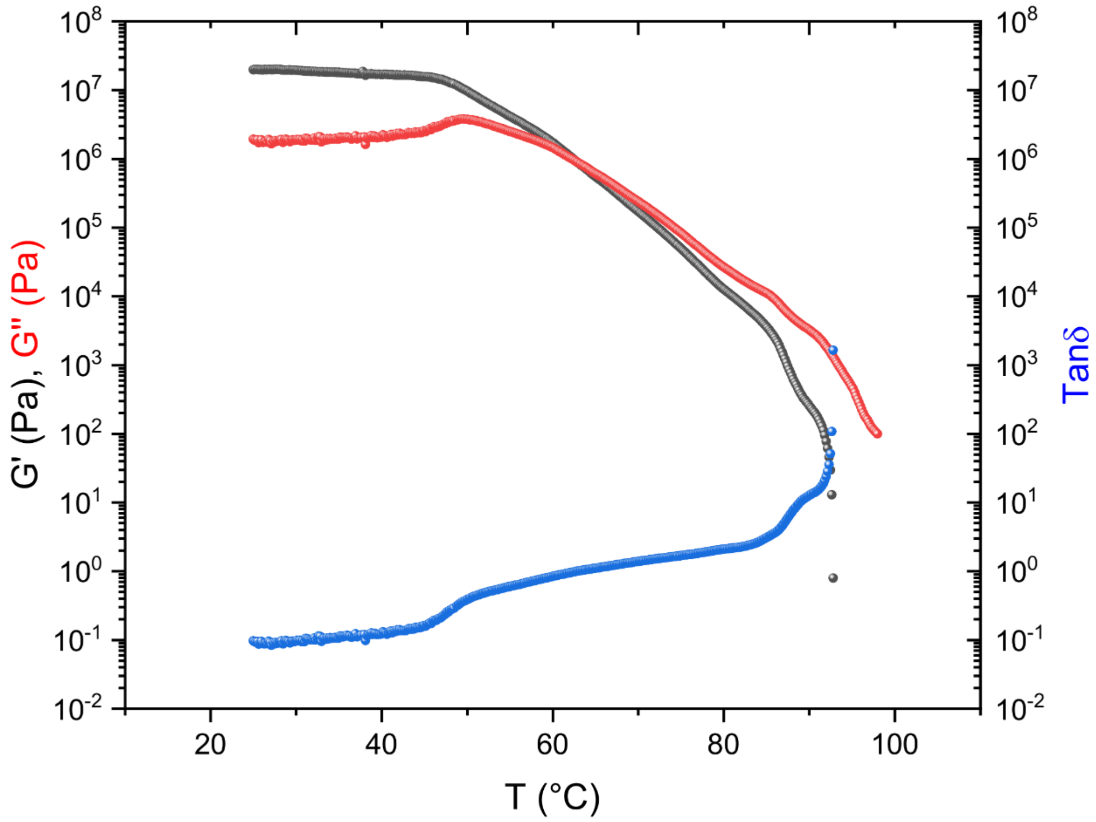

Figure S9 Dynamic Temperature Ramp Test of B30 V2000F.

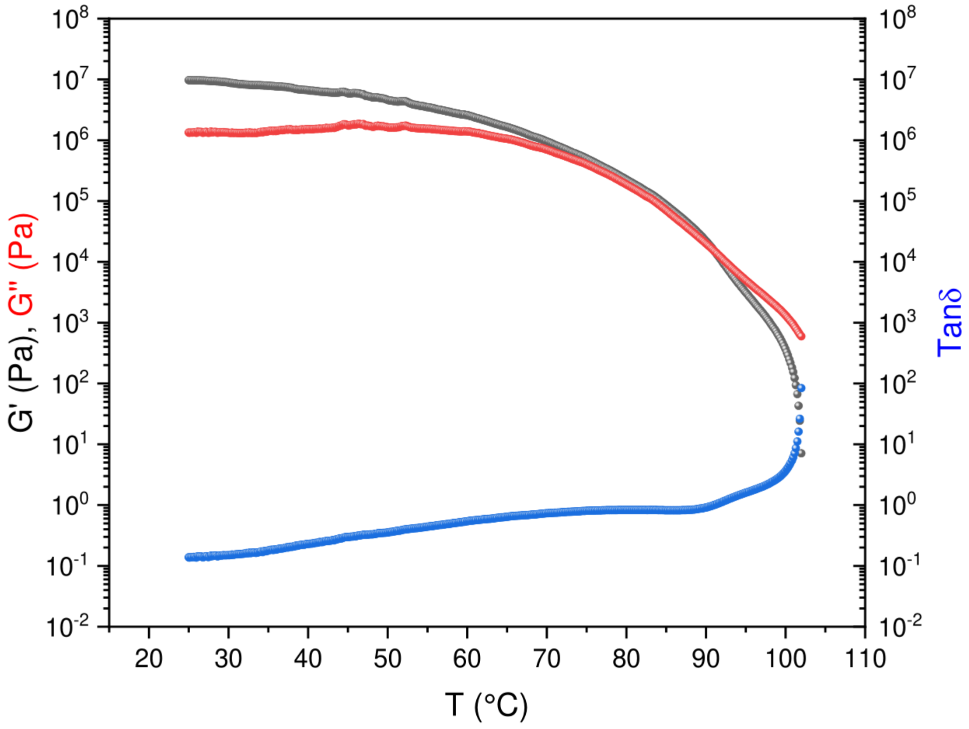

Figure S10 Dynamic Temperature Ramp Test of B31 V2000F.

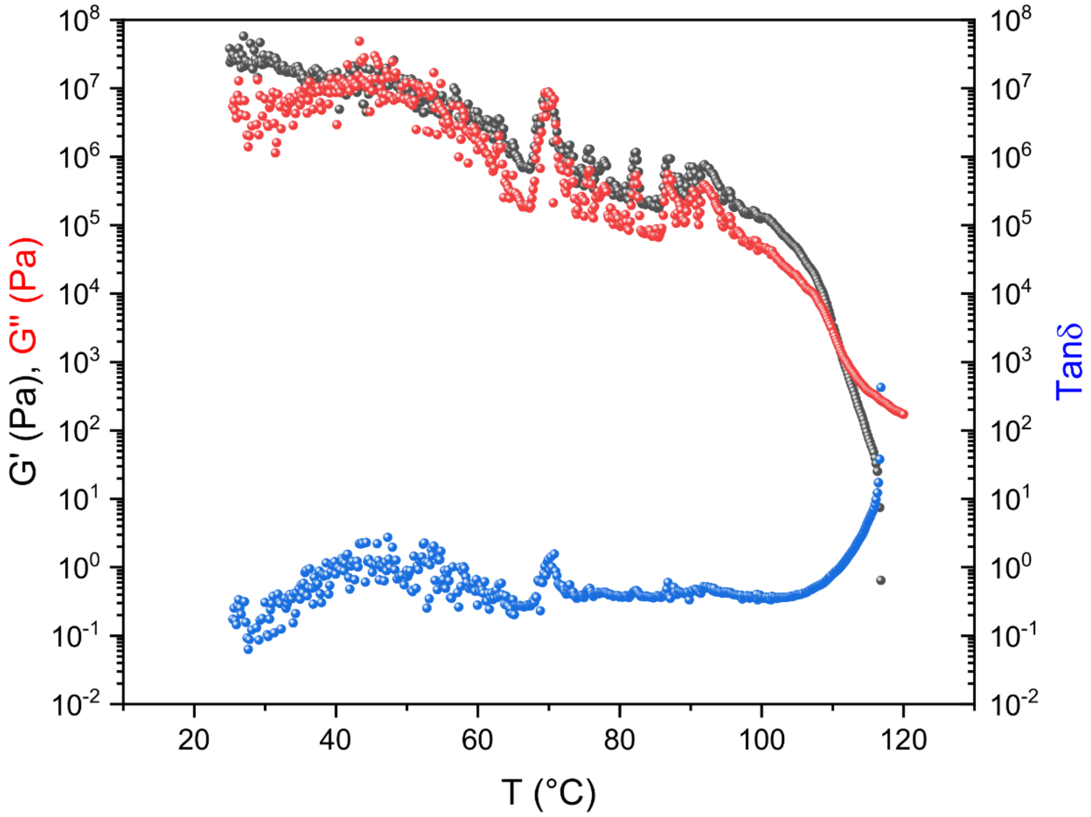

Figure S11 Dynamic Temperature Ramp Test of B31 V1000.

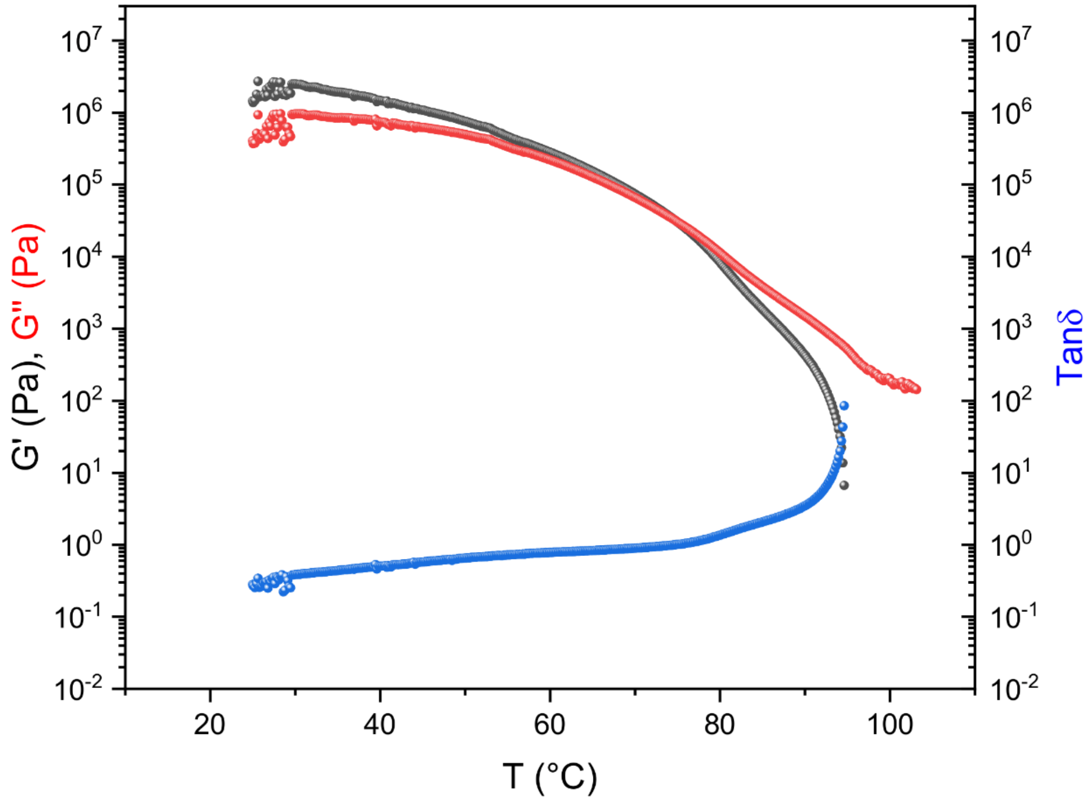

Figure S12 Dynamic Temperature Ramp Test of B32.

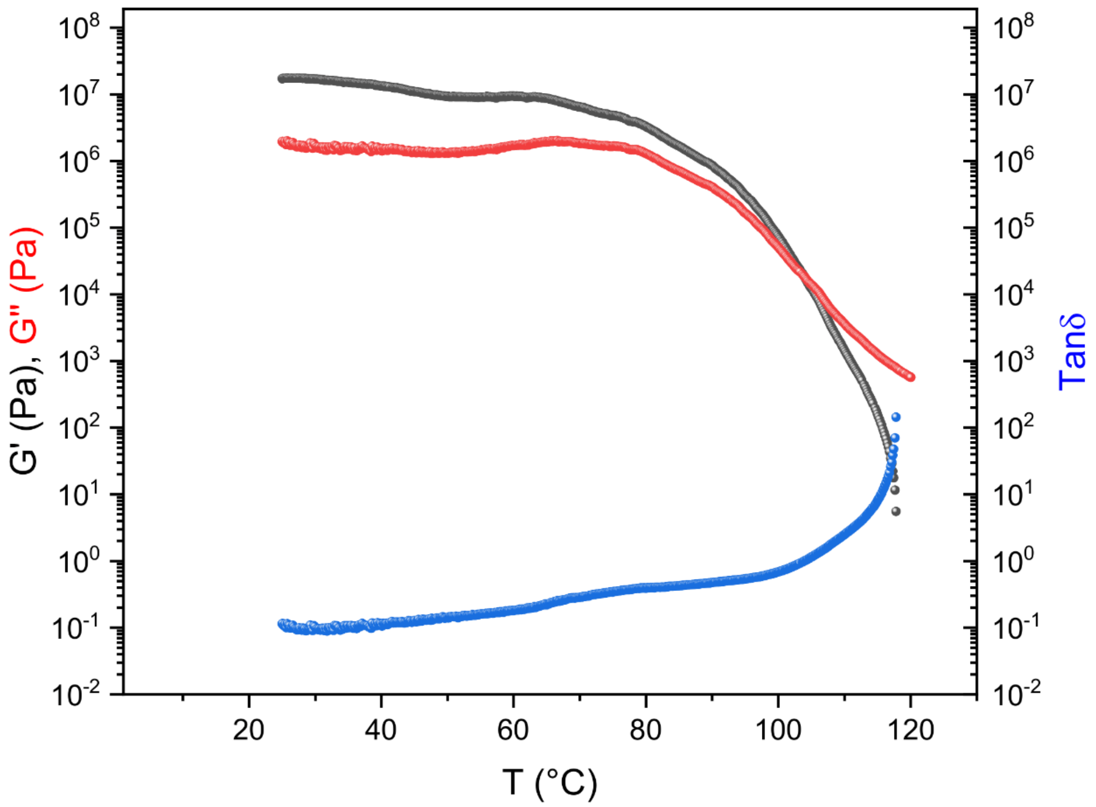

Figure S13 Dynamic Temperature Ramp Test of B33.
